# Quantitative assessment of macular function after surgery for optic disc pit maculopathy: A case report

**DOI:** 10.1097/MD.0000000000028254

**Published:** 2021-12-23

**Authors:** Wataru Inami, Yuji Yoshikawa, Masayuki Shibuya, Junji Kanno, Shunsuke Kikuchi, Yu Sakaki, Takeshi Katsumoto, Takuhei Shoji, Jun Makita, Kei Shinoda

**Affiliations:** Department of Ophthalmology, Faculty of Medicine, Saitama Medical University, Moroyama-machi, Iruma-gun, Saitama, Japan.

**Keywords:** juxtapapillary laser, retinal sensitivity, retinoschisis, serous retinal detachment, vitrectomy

## Abstract

**Rationale:**

We describe a case of optic disc pit maculopathy (ODP-M) in which vitrectomy with juxtapapillary laser (JPL) treatment led to the reattachment of retinoschisis (RS) as well as serous retinal detachment (SRD).

**Patient concerns:**

An 80-year-old man complained of distorted vision and decreased visual acuity (VA) in his left eye for 12 months.

**Diagnosis:**

We conducted quantitative functional evaluation on the area of RS and SRD using the Humphrey visual field analyzer. Fundus examination and optical coherence tomography showed SRD and RS in connection with the optic disc. The best-corrected logarithm of the minimum angle of resolution (logMAR) VA was 0.7.

**Interventions:**

The patient underwent JPL treatment combined with pars plana vitrectomy. During surgery, posterior vitreous detachment and tamponade were created with sulfur hexafluoride.

**Outcomes:**

After surgery, SRD (and subsequently RS) gradually reduced and had completely disappeared at 31 months. VA gradually improved and was 0.0 (logMAR) at 28 months. The analysis of the mean macular thickness of the central 3-mm diameter showed that the macula thickness recovered to 300 μm at 17 months postoperatively. Retinal sensitivity began to improve at 24 months postoperatively and had increased at 48 months postoperatively.

**Lessons:**

In conclusion, vitrectomy with JPL treatment for ODP-M had a favorable anatomical outcome as well as a long-term functional outcome. These findings provide useful information for clinicians who are planning a therapeutic strategy, including the choice of surgical procedure for ODP-M.

## 1 Introduction

Optic disc pit (ODP) was first described in 1882 by Wiethe^[1]^ as a congenital depression in the optic nerve head. It occurs in <1 in every 11,000 patients and can be bilateral in up to 15% of cases.^[2]^ Maculopathy associated with ODP develops in 25% to 75% of adult patients.^[2]^ In contrast, several cases of ODP maculopathy (ODP-M) without pits have been reported.^[3]^ ODP-M is congenital, and the source of the fluid and the triggers of its development are unknown.^[2]^ Nonetheless, it can occur at any age from early childhood to the eighth decade of life.^[4]^ Spontaneous resolution with good visual acuity (VA) has been reported in ∼25% of patients; however, ODP-M generally has a poor prognosis.^[2,5]^ Multiple strategies for the management of ODP-M have been suggested^[6,7]^; nevertheless, none has been established as the treatment of choice.^[8]^ Long-term anatomical and functional assessment in terms of visual acuity after reattachment has been reported.^[8–10]^ However, little information regarding the long-term outcome in the visual field in ODP-M eyes treated with pars plana vitrectomy (PPV) is available.^[10]^

We conducted a long-term quantitative functional evaluation in the areas of reattachment of retinoschisis (RS) and serous retinal detachment (SRD) using the Humphrey visual field analyzer in an eye with ODP-M that underwent PPV with juxtapapillary laser (JPL) treatment.

## 2 Case presentation

An 80-year-old man complained of distorted vision and decreased VA in his left eye for 1 year in 2015. Fundus examination and optical coherence tomography (OCT) showed SRD and RS at the level of inner nuclear layer and outer nuclear layer or Henle's fiber layer (HFL), which was connected to the optic disc. The ODP could not be clearly identified from the fundus photography and OCT images. However, the OCT image of the left eye indicated SRD and RS connected to the disc.

**Figure 1:**
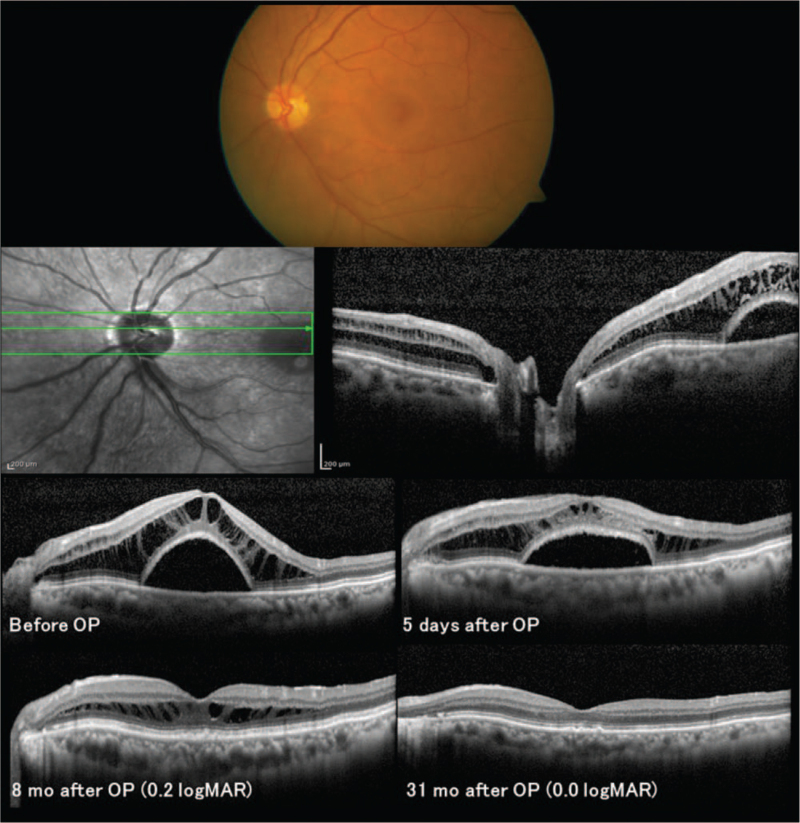
Optical coherence tomographic findings at onset and in the course of treatment. Top: Color fundus photograph at the first visit. Second row left: Infrared fundus photograph. Second row right: OCT image of the optic disc of the left eye at the initial visit showing SRD and RS connected with the optic disc. The logMAR VA is 0.7. Third row left: The OCT image of the left eye at the initial visit showing RS and SRD. Second row right: The RS and SRD have slightly decreased 5 days after pars plana vitrectomy combined with juxtapapillary laser treatment. Fourth row left: Eight months after the surgery, the SRD has completely disappeared and the RS has decreased. The logMAR VA is 0.2. Fourth row right: 33 months after surgery, the RS has completely disappeared. The logMAR VA is 0.0. logMAR = logarithm of the minimum angle of resolution, OCT = optical coherence tomography, OP = operation, RS = retinoschisis, SRD = serous retinal detachment, VA = visual acuity.

We diagnosed ODP-M with no apparent pit, as previously reported by Hedels and Krohn^[3]^ (Fig. 1). The best-corrected logarithm of the minimum angle of resolution (logMAR) VA was 0.7. The patient underwent PPV combined with JPL treatment. The JPL treatment was performed from the inferior to the temporal part of the disc (wavelength, 577 nm; spot size, 200 μm; power, 160 mW; duration, 0.1–0.5 s; total: 52 spots). During surgery, posterior vitreous detachment was created, and tamponade with sulfur hexafluoride gas was performed. After surgery, SRD (and subsequently RS) gradually reduced. The SRD had completely disappeared by 8 months; thereafter, RS had completely disappeared by 31 months (Fig. 1). At 28 months later, VA gradually improved and achieved a logMAR VA of 0.0.

**Figure 2:**
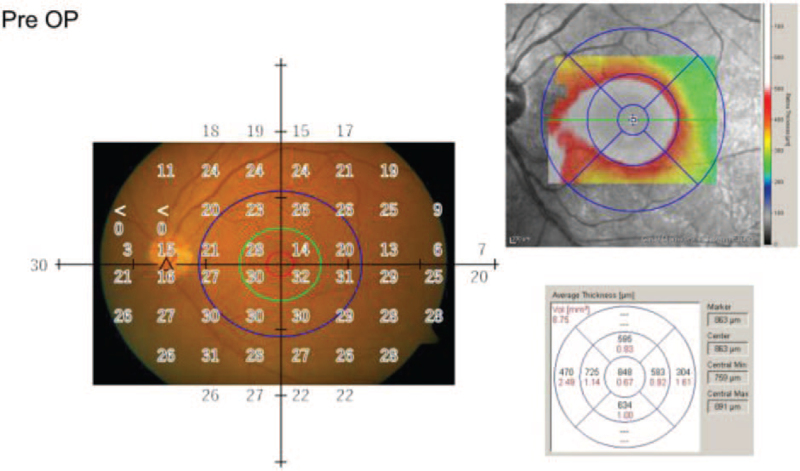
The relationship between the ETDRS grid on the mean retinal thickness map of OCT and the measurement point of the SAP before surgery. Left: The fundus picture before surgery with the central circles of different sizes and the retinal sensitivity at each point superimposed. The 1-mm-diameter central circle (red circle) includes no points in the SAP. The 3-mm-diameter central circle (green circle) corresponds to ∼10 degrees and includes 4 points in the SAP. The 6-mm-diameter central circle (blue circle) corresponds to ∼20 degrees and includes 12 points in the SAP. Top right: OCT thickness map showing ETDRS grid comprising circles with 1-, 3-, and 6-mm diameters. Bottom right: Average thickness of nine sectors corresponding to the ETDRS grid. The mean retinal thickness in the 6-mm-diameter circle could not be calculated due to the lack of measurement values of the top and bottom 1/4 sectors of the outer circumference. ETDRS = Early Treatment of Diabetic Retinopathy Study, OCT = OCT = optical coherence tomography.

**Figure 3:**
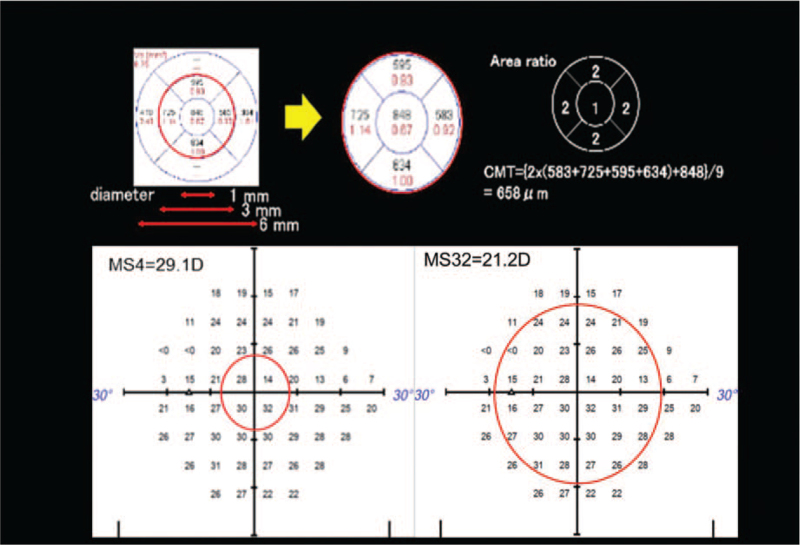
An example of the assessment on the mean retinal thickness of the central 3-mm diameter, and the corresponding mean retinal sensitivity. Top left: The mean retinal thickness of 9 areas was automatically measured by the built-in software in the OCT imaging system. The 9 areas, including the central 1 mm, are in the ETDRS chart. Top center: The mean retinal thickness of the central 1-mm area is 848 μm. Top right: The mean retinal thickness of the central 3-mm area is 658 μm, based on the following calculations. The radii of the central and outer circles are *r*_1_ = 0.5 mm and *r*_2_ = 1.5 mm, respectively. Therefore, *r*_2_ = 3r_1_. The area of the central circle (*S*_1_) and the outer circle (*S*_2_) is *S*_1_ = π*r*_1_^2^ and *S*_2_ = π*r*_2_^2^ = 9π*r*_1_^2^ = 9*S*_1_, respectively. The area of concentric circles excluding the center is *S*_2_ − *S*_1_ = 8π*r*_1_^2^ = 8*S*_1_. The area of each of the four parts in the concentric circles (e.g., temporal, nasal, superior, and inferior part) is 2*S*_1_ (i.e., Temp = Nas = Sup = Inf = 2π*r*_1_^2^ = 2 *S*_1_). The mean thickness of the central 3-mm area is {2 × (Temp + Nas + Sup + Inf) + *S*_1_}/9 = {2 × (583 + 725 + 595 + 634) + 848}/9} = 658 μm. Bottom: The mean sensitivity of the central 4 points (MS4) and 36 32 points (MS32) in the SAP were calculated as the retinal sensitivity. These points correspond to the central 3-mm area where the mean retinal thickness was measured. In addition, the mean sensitivity in the central 32 points (MS 32) was also calculated as the retinal sensitivity and corresponded approximately to the area of retinoschisis (RS). The mean retinal sensitivity was calculated for the three concentric circle areas centered on the fovea in the SAP. One circle contains 4 points, and the next size circle contains 32 points. These circles are designated as C4 and C32, respectively. The overall size in C4 corresponds to retinal circular area with ∼3-mm diameter where the mean retinal thickness was measured. The size in C32 approximately corresponds to the area of the initial RS. When calculating the average sensitivities of C4 and C32, the mean sensitivity was calculated in dB using individual test points, with each point converted to a linear scale (1/Lambert = 10 ^dB/10^; linear sensitivity) and averaged to obtain the mean sensitivity values.^[14]^ ETDRS = Early Treatment of Diabetic Retinopathy Study, OCT = optical coherence tomographic.

**Figure 4:**
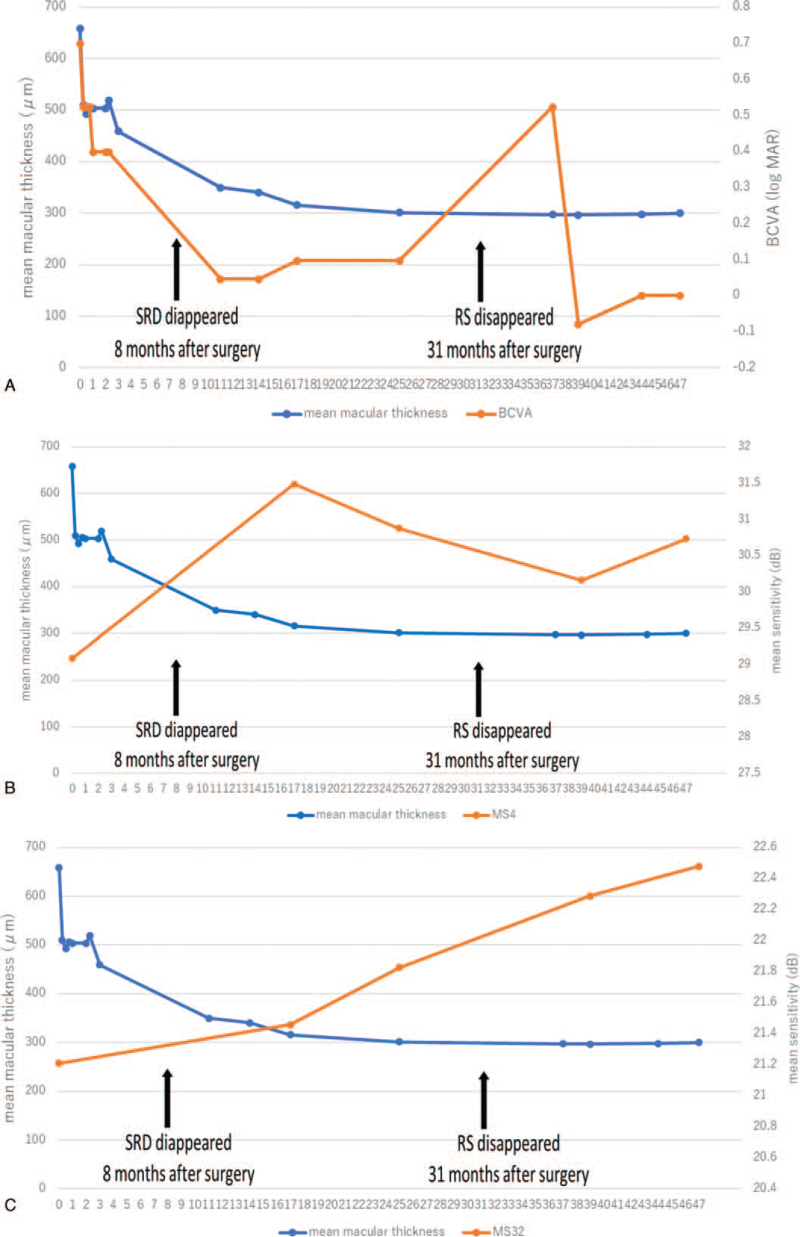
Plots of the central retinal thickness, VA, and mean sensitivity over time in an eye with optic disc pit maculopathy that underwent pars plana vitrectomy with juxtapapillary laser. (A) Changes in the mean retinal thickness of the central 3-mm circle and VA. Recovery of the mean retinal thickness and VA appear to be correlated. (B and C) Changes in the mean retinal thickness of the circles from Figure 3. The mean sensitivity (B) in the central 4 points and (C) in the central 32 points in the SAP. By 31 months postoperatively, the mean retinal thickness has gradually decreased and recovered to 300 μm when RS and SRD disappeared. It thereafter remained stable. The mean sensitivity of the central 4 points improved to a peak at 17 months after surgery when SRD vanished, but RS remained. The mean sensitivity of the central 32 points gradually and continually improved after SRD (at 8 months after surgery) and RS (at 31 months after surgery) had completely disappeared. BCVA, best-corrected VA. RS = retinoschisis, SRD = serous retinal detachment, VA = visual acuity.

We compared the mean retinal thickness and mean retinal sensitivity of the SRD and RS region. The relationship between the ETDRS grid on the mean retinal thickness OCT map and the measurement point of the standard automated perimetry (SAP) (Humphrey 24-2 Swedish Interactive Thresholding Algorithm, Carl Zeiss Meditec, Jena, Germany) is shown in Figure 2. The 3-mm-diameter central circle corresponds to ∼10 degrees and includes 4 points in the SAP. The mean macular thickness of this 3-mm-diameter circle and the corresponding mean sensitivity of SAP were analyzed (Fig. 3). The mean sensitivity for C32, which approximately corresponds to the area of the initial RS, was also analyzed. As plotted in Figure 4, the results indicated that the macular thickness and VA recovered to their normal levels as the SRD disappeared and RS gradually reduced at 17 months postoperatively. The mean sensitivity of the central 32 points (MS 32) began to improve at 24 months postoperatively and had increased at 48 months postoperatively. However, the mean sensitivity of the central 4 points (MS 4) remained stable after the SRD had disappeared and macular thickness had recovered.

**Figure 5:**
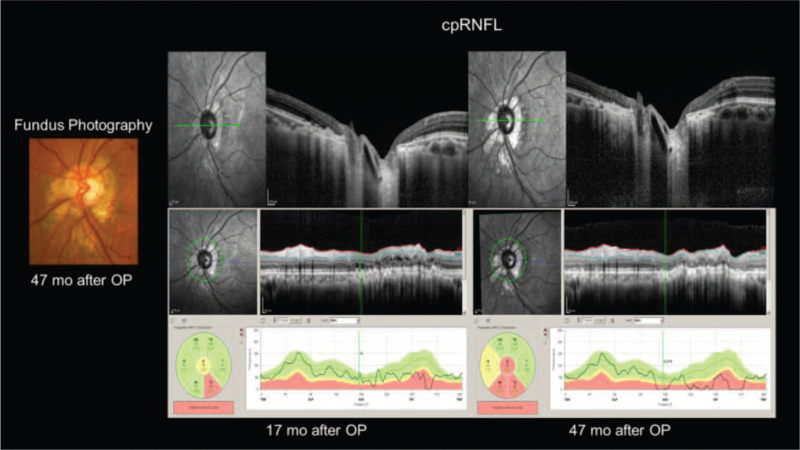
The fundus photography of the optic disc and the OCT image for the circumpapillary retinal nerve fiber layer thickness (cpRNFLT) map after JPL treatment. Left: The optic disc had a scar on the laser-treated part at 47 months after the operation. Right: The lower RNFL gradually attenuated after JPL treatment. OCT = optical coherence tomographic.

The retina inferior to the optic disc, where the JPL treatment was performed, became atrophic. The OCT image of the circumpapillary retinal nerve fiber layer thickness (cpRNFLT) map showed thinning of the cpRNFLT at the site of JPL over time (Fig. 5).

## 3 Discussion

Several surgical treatment methods for ODP-M have been proposed but remain controversial.^[6–10]^ A recent multicenter study^[7]^ reported that PPV combined with JPL treatment had similar functional and anatomical outcomes compared to PPV without JPL treatment. However, the functional evaluation was based on VA. The rationale for using JPL treatment is to establish a wall of scar tissue around the potential area of fluid entry into the retina.^[7–9]^ Nonetheless, there are few reports in which the visual field and electroretinogram were assessed in OPD-M eyes after JPL treatment.^[9,11]^ Cox et al reported that the visual field exhibited no evidence of damage to the nerve fiber layer of the retina after JPL treatment.^[11]^ The visual field showed that the blind spot was enlarged, and its shape corresponded to the pattern of laser treatment. Lei et al reported no significant defect or loss of central visual field after photocoagulation.^[10]^ In this patient, RS at the level of the inner nuclear layer immediately disappeared after surgery and VA gradually, but not substantially, improved. At 8 months postoperatively, foveal SRD had completely disappeared, and both VA and the MS4 value dramatically improved. However, the recovery of MS32, which approximately corresponded to the macular area of the RS, was limited. A previous report on central serous chorioretinopathy, using the microperimeter MP-1 (NIDEK, Gamagori, Japan), showed that eyes with resolved SRD had lower retinal sensitivity, although good VA was obtained.^[12]^

In our patient, VA recovered, and good VA was achieved with the disappearance of SRD. The MS4 value, corresponding to the central 3-mm macular area, later improved gradually. The findings of a previous cross-sectional study^[12]^ and our experience with the patient indicated that SRD was better correlated with VA than with retinal sensitivity. In addition, our experience with the patient suggested that the mean sensitivity of the foveal area could improve with long-term observation, even after SRD has disappeared.

After the recovery of VA and the reduction in the mean macular thickness, the MS32, gradually improved until 48 months after surgery, after RS had completely resolved at the level of the outer nuclear layer and HFL.

A previous case report^[10]^ followed a patient for 18 months and demonstrated that retinal sensitivity improved as the inner retinal fluid resolved, despite persistent HFL fluid; the VA then improved as the HFL fluid resolved. Our long-term observation case report demonstrated that the mean sensitivity continued to improve after the VA recovered and the RS resolved, when JPL treatment was combined with PPV. We suggest that the period of anatomical improvement was not coincident with functional improvement. The continuous recovery of MS32 reflected the retinal functional recovery of the posterior pole area, which contained the RS.

Several studies have reported high success rates of PPV combined with JPL treatment and gas tamponade.^[8,9]^ However, the considerable thinning of the cpRNFLT at the site of JPL in the current case suggests that treatment without JPL is desirable. Moreover, a multicenter case series employing PPV with a variety of adjuncts has suggested that JPL might not be necessary for successful outcomes.^[13]^

In conclusion, we presented a patient with ODP-M who underwent PPV with JPL treatment in which anatomical and functional changes were assessed with OCT and with VA and visual field, respectively. Two novel findings were illustrated:

1. VA and mean sensitivity improved long after the absorption of RS and SRD and
2. loss of mean sensitivity in the paramacular area was not clinically appreciable and the sensitivity improved until 48 months.

In conclusion, vitrectomy with JPL treatment for ODP-M initially resulted in a favorable anatomical outcome and, subsequently, a favorable functional long-term outcome. Our experience with this patient well illustrates that the mean sensitivity with SAP and VA improvement took considerable time after anatomical recovery. These findings provide useful information for clinicians who are planning a therapeutic strategy, including the choice of surgical procedure for ODP-M.

## Acknowledgments

The authors thank Editage (https://www.editage.jp) for English language editing.

## Author contributions

Conceptualization: KS. Data curation: WI, YY, MS, JK, KS. Investigation: WI, YY, MS, KS. Supervision: YS, TK, TS, JM. Writing – original draft: WI, YY. Writing – review & editing: TS, KS

**Conceptualization:** Kei Shinoda.

**Data curation:** WATARU INAMI, Yuji Yoshikawa, Masayuki Shibuya, Junji Kanno, Shunsuke Kikuchi, Kei Shinoda.

**Investigation:** WATARU INAMI, Yuji Yoshikawa, Masayuki Shibuya, Kei Shinoda.

**Supervision:** Yu Sakaki, Takeshi Katsumoto, Takuhei Shoji, Jun Makita.

**Writing – original draft:** WATARU INAMI, Yuji Yoshikawa.

**Writing – review & editing:** Takuhei Shoji, Kei Shinoda.
